# “A Cigarette a Day Keeps the Goodies Away”: Smokers Show Automatic Approach Tendencies for Smoking—But Not for Food-Related Stimuli

**DOI:** 10.1371/journal.pone.0116464

**Published:** 2015-02-18

**Authors:** Alla Machulska, Armin Zlomuzica, Dirk Adolph, Mike Rinck, Jürgen Margraf

**Affiliations:** 1 Mental Health Research and Treatment Center, Ruhr-University Bochum, Bochum, Germany; 2 Behavioral Science Institute, Radboud University Nijmegen, The Netherlands; Erasmus University Rotterdam, NETHERLANDS

## Abstract

Smoking leads to the development of automatic tendencies that promote approach behavior toward smoking-related stimuli which in turn may maintain addictive behavior. The present study examined whether automatic approach tendencies toward smoking-related stimuli can be measured by using an adapted version of the Approach-Avoidance Task (AAT). Given that progression of addictive behavior has been associated with a decreased reactivity of the brain reward system for stimuli signaling natural rewards, we also used the AAT to measure approach behavior toward natural rewarding stimuli in smokers. During the AAT, 92 smokers and 51 non-smokers viewed smoking-related vs. non-smoking-related pictures and pictures of natural rewards (i.e. highly palatable food) vs. neutral pictures. They were instructed to ignore image content and to respond to picture orientation by either pulling or pushing a joystick. Within-group comparisons revealed that smokers showed an automatic approach bias exclusively for smoking-related pictures. Contrary to our expectations, there was no difference in smokers’ and non-smokers’ approach bias for nicotine-related stimuli, indicating that non-smokers also showed approach tendencies for this picture category. Yet, in contrast to non-smokers, smokers did not show an approach bias for food-related pictures. Moreover, self-reported smoking attitude could not predict approach-avoidance behavior toward nicotine-related pictures in smokers or non-smokers. Our findings indicate that the AAT is suited for measuring smoking-related approach tendencies in smokers. Furthermore, we provide evidence for a diminished approach tendency toward food-related stimuli in smokers, suggesting a decreased sensitivity to natural rewards in the course of nicotine addiction. Our results indicate that in contrast to similar studies conducted in alcohol, cannabis and heroin users, the AAT might only be partially suited for measuring smoking-related approach tendencies in smokers. Nevertheless, our findings are of special importance for current etiological models and smoking cessation programs aimed at modifying nicotine-related approach tendencies in the context of a nicotine addiction.

## Introduction

Tobacco smoking is one of the most common health-impairing behaviors, leading to serious health consequences and an increased risk of premature death. Despite the awareness about these hazardous consequences, smokers continue to express such maladaptive pattern of behavior which represents a serious challenge for conventional smoking cessation programs.

According to dual process models of addiction, two highly interacting but otherwise opponent systems need to be considered in order to understand why individuals display maladaptive addictive behavior: An appetitive, approach-oriented system which induces fast, effortless, automatic impulsive-like reactions, and an executive system which induces rather slow, effortful, reflective and controlled reactions [1–4]. Addiction can be considered a result of an imbalance, instead of cooperation, between these two systems. In particular, Wiers and colleagues applied the dual process theory to the more precise context of addiction in order to make predictions on whether or not addictive behavior (i.e. smoking) will be initiated and/or continued [4]. According to Wiers, smoking and/or repeated confrontation with nicotine and nicotine-related stimuli is supposed to strengthen the appetitive system, which leads to the development of automatic approach tendencies to smoking-related and nicotine-related stimuli. These automatic approach tendencies toward smoking-related stimuli have been strongly implicated in the maintenance of smoking behavior as well as in the likelihood of relapse after successful smoking cessation treatment [5].

Accordingly, while conventional cessation programs and psychotherapeutic interventions so far predominantly focus on improving the patient’s understanding of the harmful consequences of smoking in order to inhibit future addictive behavior, recent approaches also emphasize the importance of measuring and changing automatic, unconscious processes in order to modify the individual’s tendency to initiate and continue addictive impulses in the long-term [6].

In recent years, a great effort has been invested into the development of tools suited to access automatic drug-approach behaviors which are usually devoid of intentional or cognitive control [6]. Until recently, only a few valid and reliable methods for the assessment of approach and avoidance tendencies in the course of physical addictions to substances exist and it remains unclear to which extent these tasks really tag implicit or automatic processes [7]. Recently, Rinck and Becker [8] developed the Approach-Avoidance Task (AAT) suitable for both the assessment and modification of automatic approach behavior in the course of alcohol dependency [9–12], cannabis use [13] and heroin abuse [14]. During the AAT, participants view pictures on a computer screen and subsequently pull or push a joystick as fast as possible in response to a content-irrelevant feature such as picture orientation or format. Upon a pull-movement, the picture size is increased, whereas upon a push-movement the picture size is decreased, creating a sense of approach or avoidance, respectively (“zooming feature”) [8]. The basic idea behind the AAT is that if reaction times vary in relation to image content, they are biased by the automatic evaluation of the content [15]. In particular, faster pulling than pushing of a picture indicates an automatic approach tendency, whereas faster pushing than pulling indicates an automatic avoidance tendency for this picture. Thus, the AAT is an ecologically valid method to assess and measure automatic approach or avoidance behavior in the laboratory setting. Most importantly, the experimental manipulation of existing automatic biases toward drug-associated cues by specifically designed training programs based on the AAT can be effective in decreasing addictive behavior and reducing the susceptibility to relapse [16], [12]. For example, in a recent study in hazardous drinkers, existing automatic action tendencies to approach alcohol could be changed successfully using a newly developed training version of the alcohol AAT. Furthermore, the change in automatic action tendencies was found to be clinically relevant, since heavy drinkers in the avoid-alcohol condition drank less beer during the subsequent taste test [11]. Similarly, Wiers et al. [12] demonstrated that patients trained to avoid alcohol exhibit less relapse at one-year follow up.

To date, evidence for automatic approach biases for nicotine-related cues in smokers is rather rare [17]. Thus, our first aim was to confirm and replicate that the AAT can also be used to assess approach-avoidance biases toward smoking-related stimuli in smokers. Based on previous investigations, we hypothesize that compared to non-smokers, smokers will display an automatic approach bias for smoking-related pictures, i.e. react by pulling nicotine-related pictures faster than pushing these pictures, as compared to a category of neutral, shape-matched pictures [17].

Mounting evidence from neuroimaging studies suggests that the progression of addiction leads to adjustments in brain reward mechanisms resulting in an increased sensitivity to conditioned drug stimuli [18]. While addicted individuals become more and more hypersensitive to drug-related stimuli as addiction increases, they concomitantly exhibit decreased sensitivity to natural rewards such as food [19]. For instance, adolescent light smokers show decreased activations in multiple brain reward areas including the insula and frontal regions when viewing food versus neutral images [19]. Furthermore, nicotine administration in normal-weight never-smokers alters food-cue reactivity in brain regions previously implicated in reward and food-intake, such as the right hypothalamus and the basal ganglia [20]. Finally, by using an incentive delay task, Peters and colleagues [21] demonstrated that compared to non-smokers, smokers show decreased neural activation in the ventral striatum during anticipation of a food reward. While these data suggest that smoking and nicotine administration is associated with changes in responding to natural rewards (in particular food rewards) in favor of smoking-related cues due to alterations in brain reward circuits, evidence so far stems exclusively from neuroimaging studies. In fact, to date no behavioral evidence is available to the question whether smokers also show alterations in automatic approach tendencies when subjected to natural rewards such as highly palatable food images. Thus, our second aim was to examine whether such alterations exist at the behavioral level, and whether they can be measured with the AAT. We assumed that compared to non-smokers, smokers would show a decreased automatic approach bias to natural rewards (i.e. pictures of highly palatable food) in the AAT.

Given that dual process models postulate that smoking behavior is maintained by strengthened impulsive systems and weakened reflective systems, we questioned whether smokers would perform similarly on measures which reflect these two opponent processes. Therefore, in addition to performance in the AAT, we assessed smokers’ and non-smokers’ subjective attitudes toward smoking, using a self-report questionnaire. By doing so, we sought to determine whether specific predictions on the magnitude of approach behavior toward smoking-related pictures could be made based on general subjective attitudes toward smoking.

## Materials and Methods

### Subjects

A total of 92 smokers and 51 non-smokers were recruited from the student population at the Ruhr-University Bochum (Germany) through advertisement via board announcements and flyers. Participants were naïve to the experimental hypothesis and test conditions. Smokers were included if they had smoked for at least 12 months at least 6 cigarettes per day. The group of non-smokers consisted of individuals who had smoked less than 10 cigarettes in their entire lifetime. Subjects received either money (10euro/h) or course credit for participation. Exclusion criteria for all participants were a history of major medical or psychiatric disorders.

### Ethics statement

The study was approved by the local Ethics Committee of the Ruhr-University Bochum and was conducted in accordance with the Declaration of Helsinki. Each participant provided a written informed consent to the experimental procedure prior to the inclusion in our study.

### Questionnaires and clinical measures

Nicotine use was assessed with the Fagerström Test for Nicotine Dependence (FTND) [22]. The FTND is a widely used self-report measure, consisting of six items aimed to capture the degree of nicotine dependence (e.g. “How many cigarettes per day do you smoke?”) on a range from 0 to 10. Scores between 0 and 2 signal no or very weak dependence, scores between 3 and 4 weak dependence, a score of 5 moderate dependence, scores between 6 and 7 strong dependence, and scores between 8 and 10 very strong nicotine dependence.

There is considerable evidence that smoking is associated with increased depressive-like behavior as well as changes in stress- and anxiety levels [23–24]. To control for differences in depression, stress and anxiety levels in smokers and non-smokers, and to measure possible influences of these variables on performance in the AAT, all participants completed the Depression-Anxiety-Stress-Scale 21 (DASS 21G) [25]. The DASS 21G is a self-report measure for assessing the three negative emotional states: depression, anxiety and stress [25]. Each of the three scales is measured through 7 items, resulting in a total of 21 items. The items consist of statements referring to the past week, ranging from 0 (did not apply to me at all) to 3 (applied to me very much or most of the time). Thus, scores for each scale range from 0 to 21. Cut-off scores for increased risk of further mental disorders and considerable high disturbance in matters of depression and stress are scores above 10, cut-off scores for anxiety are scores above 6 [26].

Positive and negative attitudes for smoking behavior were evaluated by asking each participant to rate a set of eight items which describe smoking behavior in terms of positive and negative adjectives: e.g. good-bad, healthy-unhealthy, sexy-unsexy, pleasant-unpleasant, harmless-harmful, sociable-unsociable, ugly-glamorous and calming-stressful. The items in this measure were based on the ones used by Swanson and colleagues [27]. Scales ranged from 1 to 7 with a score of 1 indicating an extremely positive attitude toward smoking and a score of 7 indicating an extremely negative attitude. To make the scale more intuitive, we transformed it, resulting in a modified range between +3 and −3 with a score of +3 meaning an extremely positive, −3 meaning an extremely negative and 0 meaning a neutral attitude toward smoking.

### Assessment of automatic approach tendencies with the Approach-Avoidance-Task

**Figure 1:**
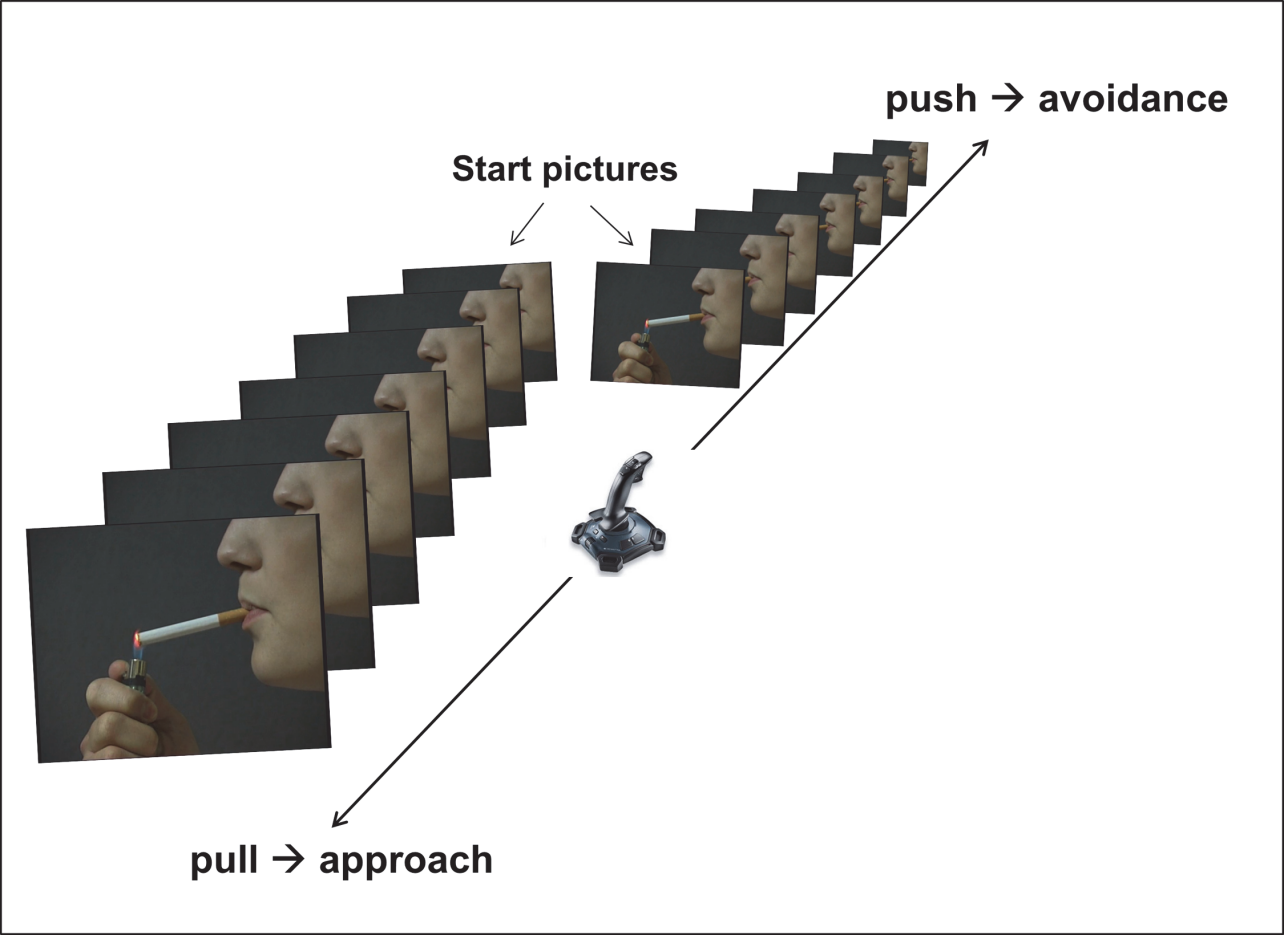
Schematic demonstration of the trials used in the AAT for nicotine-related pictures. Pulling the joystick increases the picture size, while pushing the joystick decreases the picture size.

An adapted version of the Approach-Avoidance-Task was used to assess automatic approach biases toward smoking-related and food-related cues in smokers and non-smokers. The AAT was a computer-based task during where different pictures were displayed on a 13-inch screen. The images were either rotated 3° to the left or to the right. A joystick was connected to the computer, and participants were instructed to pull pictures rotated to the left and to push pictures rotated to the right as quickly and accurately as possible. In addition, pulling the joystick increased the picture size, while pushing the joystick decreased the picture size (see Fig. 1 for an exemplary AAT-trial). Consequently, back-and-forth joystick movements resulted in corresponding grow-and-shrink changes in picture size. This dynamic zoom effect creates a sense of approaching or avoiding the stimulus and disambiguates the task [8]. Before the actual AAT started, all participants completed 12 practice trials with unrelated pictures depicting balloons and a motorcycle. Thereafter, the AAT was performed during which 192 trials were presented in quasi-random order (at most three equal rotations and image categories in a row). The first 96 trials were followed by a short break to avoid loss of concentration.

### AAT stimuli

We used four different picture categories in the AAT: a) smoking-related pictures, b) pictures of toothbrushes, c) food pictures and d) neutral pictures. Smoking-related and shape- and color-matched pictures of persons brushing their teeth were provided by Stippekohl and colleagues, who validated these pictures in a previous neuroimaging study [28]. Nicotine-related items contained images depicting smoking-related actions from the commencement of smoking-behavior (i.e. person lighting a cigarette), but not actions from the terminal stage of smoking (i.e. person stubbing out a cigarette). This was done because Stippekohl et al. [28] previously used functional neuroimaging contrasts to show that pictures depicting the initial phase of smoking are more suitable to trigger activation in brain addiction networks and that smokers rated such stimuli more positively compared to stimuli of the terminal phase of smoking. Pictures of toothbrushes and persons brushing their teeth were also derived from the pictures previously used by Stippekohl et al. [28] and served as a neutral category for the comparison with smoking-related pictures. Pictures of high-palatable foods such as pizza, burgers or ice cream and corresponding shape- and color-matched neutral pictures were taken from the International Affective Picture System (IAPS) [29]. Each of the categories contained 8 images presented in a quasi-random order during the AAT task. The order of picture presentation was identical for both smokers and non-smokers.

### Experimental Procedure

As a part of the instructions, all participants were explicitly informed that they had to refrain from eating food and drinking beverages apart from water, at least 1 hour prior to the testing. Additionally, participants were instructed to refrain from alcohol 24 hours prior to the experimental testing. To achieve a comparable level of craving among smokers, all smokers were further instructed to abstain from smoking for one hour prior to the testing. After arrival at the laboratory, all participants received further instructions on the experimental procedure, and read and signed an informed consent form. Thereafter, participants were asked to rate their level of craving, completed the FTND and indicated their attitude toward smoking. Subsequently, the AAT was performed.

### Data preparation and statistical approach

Before calculating the AAT scores, all trials during which participants showed a false reaction (i.e. pushing/pulling instead of doing the opposite) were excluded [13]. AAT-bias scores were calculated by subtracting median RTs for pulling a picture from median RTs for pushing a picture (*median RTpush – median RTpull*) according to the procedure used by Rinck & Becker [8]. Thus, positive AAT-scores indicate an approach bias and negative AAT-scores an avoidance bias for the particular image category. AAT-scores near zero indicate neither approach nor avoidance. Participants with a high number of incorrect reactions during the AAT (>25% of incorrectly performed reactions) were excluded from the analysis because high error percentages suggest a lack of concentration and/or motivation during the AAT task [10]. In sum, 4 participants (2 smokers and 2 non-smokers) were excluded due to extremely high error rates. Hence, data from 90 smokers and 49 non-smokers were included in the final analyses.

Between-group differences in the AAT-bias scores were analyzed by means of a repeated-measures ANCOVA with smoker vs. non-smoker as between-subjects factor, image type (nicotine-related pictures vs. pictures of toothbrushes vs. food pictures vs. neutral pictures) as within-subjects factor, and specific variables where smokers and non-smokers differed and which could affect AAT performance as covariates. Significant group differences and/or group × stimulus interactions were further analyzed by one-sample and two-sample t-tests. To correct for multiple comparisons, the Bonferroni correction was used. The level of significance was considered p < .05 (two-tailed).

To test for a possible link between self-reported smoking attitudes and nicotine-approach/avoidance biases, we used a hierarchical regression analysis with smoking attitudes as the predictor variable and potential biases in the AAT as the criterion.

## Results

### Sample characteristics

Table 1 presents the demographic description of the smoking and non-smoking group. While there was no difference in gender distribution between groups (*F*(1,138) = 2.77, *p* = .1), the groups did differ in several other control variables. For instance, smokers were significantly older than non-smokers (*F*(1,137) = 11.43; *p* < .001) and they reported significantly more depression (*F*(1,137) = 7.72; *p* < .01), anxiety (*F*(1,137 = 6.75; *p* < .01) and stress (*F*(1,137) = 10.96; *p* < .001), as measured with the DASS 21G. However, the mean scores in the DASS did not exceed the cut-off scores for high emotional overload in smokers or non-smokers (see Table 1). Given that the groups differed in age and DASS scores, we included these variables as covariates in our subsequent analyses. The overall FTND-score for smokers (*M* = 3.38; *SD* = 2.3) suggested a weak dependence according to the classification of Heatherton et al. [22]. Prior to the AAT, smokers reported a medium level of craving (*M* = 2.58; *SD* = 1.43). As expected, no craving was reported by non-smokers (*M* = 0). Accordingly, a significant group difference in self-reported craving was evident between smokers and non-smokers (*F*(1,137) = 160.6; *p* < .001).

**Table 1:** Mean sample characteristics and performance in the AAT.

	**Group**			
	**Smokers**	**Non-Smokers**	**Statistical value F**	**p-value**
**N**	90	49	-	-
**Age**	26.63 (6.34)	23.33 (3.44)	11.43	<.001
**Gender (%female)**	44%	59%	2.77	.1
**Depression**	3.19 (3.48)	1.71 (1.68)	7.72	<.01
**Anxiety**	3.09 (2.8)	1.94 (1.73)	6.75	<.01
**Stress**	7.14 (4.3)	4.81 (3.14)	10.96	<.001
**FTND-Score**	3.38 (2.3)	-	-	-
**0–10 smoked cigarettes/day**	38%	-	-	-
**11–20 smoked cigarettes/day**	51%	-	-	-
**21–30 smoked cigarettes/day**	8%	-	-	-
**>31 smoked cigarettes/day**	3%	-	-	-
**Craving**	2.58 (1.41)	0	160.6	<.001
**Error rate in AAT (%)**	8% (5)	11%(6)	10.48	<.01
**Pull**	619 (118)	590 (97)	2.17	.14
**Push**	621 (110)	599 (91)	1.43	.23
**Global smoking attitude**	−.16(.54)	−1.87(.67)	271.04	<.001

*Note*. N = number of participants; FTND-Score = Score in Fagerström Test for Nicotine Dependence; standard deviations are given in parentheses; variables were analyzed with one-way-ANONA, (*F*1,137); all p values are two-tailed.

### Automatic action tendencies

Table 2 summarizes mean AAT-bias scores per image category and group. All dependent variables were distributed equally (*ps* > .20, Kolmogorov-Smirnov test of goodness of fit) and no violation of the homogeneity-of-variances assumption was found (*ps* > .1, Levene-Test). Finally, the sphericity assumption was not violated (*χ^2^* = 2.7, *p* = .75, Mauchly-Test). Statistical analyses revealed no group differences in median RTs for pushing or pulling pictures, averaged across all four image categories (for pushing: *F*(1,137) = 1.9, *p* = .27, One-Way-ANOVA; for pulling: *F*(1,137) = 2.21, *p* = .14). However, smokers made significantly fewer mistakes in the AAT than non-smokers (*F*(1,138) = 10.48, *p* < .01). Hence, we also controlled for percentage of correct reactions in the subsequent analysis.

**Table 2:** Mean AAT-bias-scores for smokers and non-smokers per image type.

	**Group**					
	**Smokers**			**Non-smokers**		
**AAT-Score**	***M***	***SD***	***SE***	***M***	***SD***	***SE***
**Nicotine-related images**	25	75	8	10	62	9
**Pictures of toothbrushes**	−12	64	7	−17	73	10
**Food images**	−1	59	6	21	56	8
**Neutral images**	0	69	7	11	57	8

*Note*. M = mean; SD = standard deviation; SE = standard error; a positive AAT-score indicates an approach bias, a negative AAT-score an avoidance bias.

Differences in the AAT-bias scores were analyzed with a mixed ANCOVA, in which we entered age, depression, anxiety, stress and error rates as covariates. As expected, there was neither a main effect for group (*F*(1,130) = .65; *p* = .42; *η^2^* = .01), nor a main effect for image type (*F*(3,128) = 1.45; *p* = .23; *η^2^* = .03). We further analyzed possible interactions between the covariates and the AAT scores. However, there were no significant interactions between image type and age (*F*(3,128) = 1.21; *p* = .31; *η^2^* = .03), depression (*F*(3,128) = .21; *p* = .89; *η^2^* = .01), stress (*F*(3,128) = .5; *p* = .69; *η^2^* = .01), anxiety (*F*(3,128) = 1.02; *p* = .39; *η^2^* = .02) or error rates (*F*(3,128) = 2.09; *p* = .11; *η^2^* = .05), indicating that differences in DASS scores, age or percent of correct reactions did not differentially affect approach or avoidance behavior in the AAT in the respective groups.

**Figure 2:**
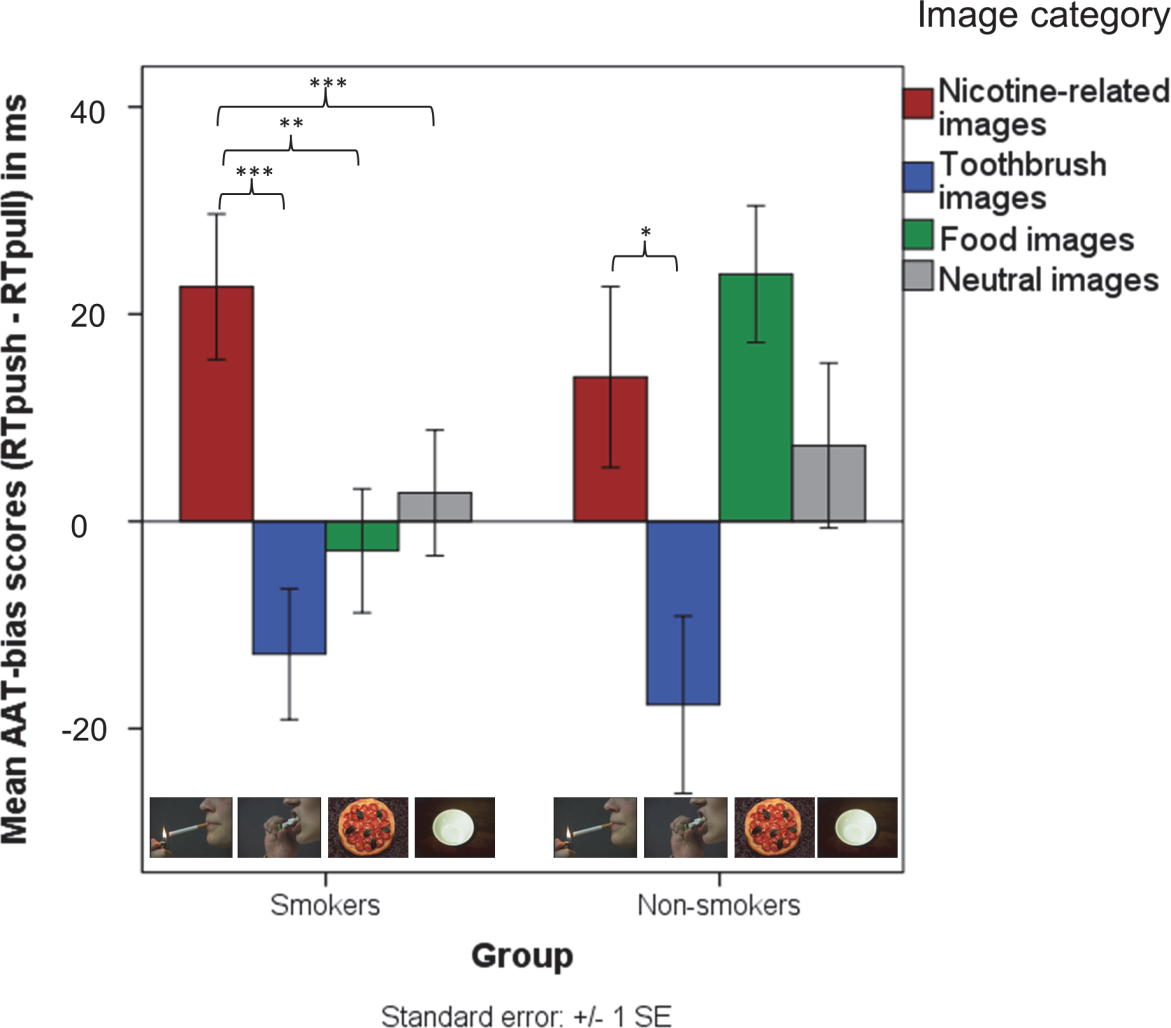
Mean AAT-bias scores for nicotine-related images, images of toothbrushes, food images and neutral images, in smokers and non-smokers. A positive score indicates an approach tendency, a negative an avoidance tendency. Error bars correspond +/− 1 standard error. *p < .05; **p < .01; ***p < .001.

In line with our hypotheses, the multivariate interaction between image type and group was significant (*F*(3,128) = 3.26, *p* < .05, *η^2^* = .07) (see Fig. 2). Further analyses revealed that smokers showed a significantly larger approach tendency toward nicotine-related pictures than to pictures of toothbrushes (*t*(89) = 5.73, *p* < .001), food pictures (*t*(89) = 3.54, *p* < .01) and neutral pictures (*t*(89) = 4.22, *p* < .001). Non-smokers’ bias scores for nicotine-related images did not deviate significantly from the bias scores for other image types (*ps* > .9), except for pictures of toothbrushes (*t*(48) = 2.91; p < .05). Hence, non-smokers showed only an approach bias for nicotine-related pictures when compared to pictures of toothbrushes. Although the approach-biases for nicotine-related images were not significantly different between smokers and non-smokers (*t*(137) = 1.17; *p* = .24), there was a trend toward a significant group difference in approach tendency toward food-related pictures (*t*(137) = 1.8, *p* = .075). Fig. 2 illustrates the effects defined above and depicts smokers’ and non-smokers’ approach and avoidance biases for the particular image categories (see S1 Fig. in the section “Supporting Information” for a distribution of individual AAT-Bias scores).

### Attitude toward smoking

Non-smokers rated their attitude toward smoking as negative on all smoking-relevant semantic differential items (all *ps* < .01) except on the items “sociable” and “glamorous” (*ps* > .26). In contrast, smokers rated smoking as bad (*t*(89) = 6.62, *p* < 001), unhealthy (*t*(89) = 28.89, *p* < .001) and harmful (t(89) = 17.72, p < 001), but also classified smoking behavior as pleasant (*t*(89) = 3.67, *p* < .001), sociable (*t*(89) = 9.58, *p* < .001) and calming (*t*(89) = 11.7, *p* < .001). Furthermore, smokers rated all eight items together as more positive than non-smokers did (*ps* < .023) (see S1 Table in the “Supporting Information” section).

A composite score was obtained by calculating the average of all eight scores per person which served as a global attitude toward smoking score. As expected, smokers’ attitude toward smoking was more positive than non-smokers’ (*F*(1,138) = 271.04, *p* < .001; see Table 1). However, both non-smokers’ (*t*(48) = −19.69) *and* smokers’ (*t*(89) = −2.82) attitude toward smoking was negative, deviating significantly from zero (*ps* < .004).

We used hierarchical multiple regression analysis to examine whether smoking attitudes could predict nicotine-related approach-avoidance tendencies in smokers and non-smokers, in addition to the demographic variables age and gender, and the smoking-related variable FTND-score. Prior to the main analyses we ensured that assumptions of normality, linearity, homoscedasticity and no multicollinearity were met. The histogram and probability plot of the residuals did not indicate that any of the regression assumptions were violated neither in the smokers, nor in the non-smokers group. The collinearity statistics confirm that multicollinearity is not a problem in our case (largest *VIF* for smokers = 1.09; largest *VIF* for non-smokers = 1.05). For each group, age and gender were entered at step 1, explaining a total of 1.2% of variance in the nicotine-bias-score in smokers and 4.7% of variance in the nicotine-bias-scores in non-smokers (both non-significant: *ps* > .33). In the group of smokers, the FTND-Score was entered at step 2, explaining a total variance of 1.5% (*p* = .6). Finally, the variable “attitude toward smoking” was entered in the analysis. The total variance explained by the model was 1.7% in smokers (*p* = .7) and 5.3% in non-smokers (*p* = .6). These results indicate that in both groups, the models could not predict nicotine-related approach-avoidance tendencies. Hence, it may be suggested that the nicotine bias is rather independent of direct measures of self-reported smoking attitudes.

## Discussion

The current study was designed to examine automatic approach tendencies for nicotine- and food-related stimuli in smokers and non-smokers. We hypothesized that an approach bias toward nicotine-related stimuli as compared to other stimuli can be detected via the AAT in smokers, but not in non-smokers. Although we found an automatic approach bias for nicotine-related stimuli in smokers compared to all other stimulus categories, a rather unexpected finding was that – to a lesser extent – non-smokers also showed an approach bias toward nicotine-related stimuli. These data suggest, that although the AAT might be partially suited to detect an approach bias toward nicotine-related stimuli in the course of nicotine addiction, such an approach bias is less clear-cut than in the context of other addictions and might be modulated by other factors such as the heaviness of smoking and/or duration of smoking deprivation. We further aimed to detect group differences in approach biases toward natural rewards. Given that previous neuroimaging studies showed a decreased sensitivity to natural rewards such as food in smokers, we hypothesized that such alterations in reward sensitivity can also be detected at the behavioral level by means of the AAT. Consistent with our hypothesis, an approach bias for food-related stimuli could only be detected in non-smokers, but not in smokers. Hence, we herein provide first behavioral evidence for specific alterations in automatic approach tendencies towards natural rewards in smokers. In the following sections, we discuss both etiological as well as therapeutic implications of these findings in the context of nicotine addiction.

One important finding of the study was that smokers exhibited a specific approach bias for nicotine-related stimuli which did not generalize to other pictures such as pictures of toothbrushes, food or neutral images. However, between-groups analysis failed to detect a difference in nicotine-approach biases between smokers and non-smokers. Hence, these data suggest that the AAT might not be sensitive to detect an approach bias for nicotine-related stimuli as previously shown in the context of alcohol, cannabis and heroin addiction [10], [13–14]. Interestingly, two recent studies successfully demonstrated an approach bias for nicotine-related stimuli by means of the AAT [17], [30], suggesting that methodological differences and/or differences in sample characteristics affect the magnitude of the nicotine-approach bias in smokers. For instance, the relatively weak approach bias toward nicotine-related cues in smokers might be attributed to the level of heaviness of smoking in our sample. In fact, the inclusion criteria for smokers in our study were rather low, which was also reflected in the FTND scores. The smoking group consisted of relatively moderate smokers with a mean FTND score of 3.4 (SD = 2.3). Compared to that, Wiers et al. [17] demonstrated a nicotine-approach bias in smokers with a mean FTNA score of 5.1 (SD = 1.2), but not in a sample of never smoking individuals or ex-smokers. Hence, in future studies it would be interesting to examine whether the approach bias is dependent on the heaviness of smoking. Another important variable which might have affected group differences in the approach bias for smoking-related pictures is the duration of smoking deprivation. In particular, we instructed smokers to abstain from tobacco smoking at least one hour prior to the testing to induce comparable levels of craving among smokers. In contrast, Wiers and colleagues [17] deprived smokers for at least two hours and found a stronger nicotine-approach bias in smokers compared to never-smokers or ex-smokers by using the AAT. Similarly, Watson et al. [30] used an even longer deprivation period (approximately 13 hours) and found a substantial approach bias for nicotine stimuli in smokers. The approach bias in smokers, however, increased after subsequent nicotine consumption. Thus, although these studies suggest that duration of the deprivation period might indeed have an impact on the approach bias in smokers, certainly more research is needed to identify all potential factors affecting the approach bias toward nicotine-related stimuli. Finally, it is conceivable that genetically determined differences in the magnitude of nicotine-related approach bias exist in smokers as previously shown in heavy drinkers [10]. For instance, Wiers et al. [10] demonstrated that the approach bias in the AAT in heavy drinkers is related to a variation in the OPRM1 gene. The genetic variation in the OPRM1 gene has previously been implicated in the processing of the rewarding effects of alcohol and other drugs [10]. Interestingly, Wiers et al. showed that among heavy drinking individuals, only carriers of the g-allele of the OPRM1 gene showed an automatic approach tendency for alcohol-related and other appetitive stimuli (pictures of soft drinks) in the AAT, while non-carriers did not show a similar approach bias. Hence, it would be valuable to examine whether genetically determined inter-individual variations in the tendency to approach or avoid nicotine-associated stimuli exist in smokers as well.

A rather unexpected finding in our study was that similar to smokers, non-smokers also exhibited an approach bias for smoking-related pictures. The exact mechanism underlying an approach bias toward smoking-related cues in non-smokers remains elusive and might be due to reasons which are not related to addictive processes. Although such an effect was not expected, biases for smoking cues in non-smokers have also been reported elsewhere [32]. For instance, it is possible that non-smokers showed an approach bias toward smoking-related pictures because of the particular valence of the stimuli used (see also [33]). In a similar study using the Stimulus-Response-Compatibility-Task (SRCT), Bradley and colleagues [32] assessed approach tendencies in smokers and non-smokers for pleasant and unpleasant nicotine-related cues. Although a significant difference between smokers’ and non-smokers’ approach bias for unpleasant nicotine-related images emerged, such group differences were not evident when pleasant nicotine-related cues were used. In fact, when using pleasant nicotine-related images in the SRCT, non-smokers showed a similar approach bias for this content category as smokers. In the present study, we used the picture set previously used by Stippekohl et al. [28]. Here, images depict the initial phase of the smoking ritual which is suggested to be perceived as more pleasant than images depicting the terminal phase of the smoking ritual [28]. Thus, similar to the findings by Bradley et al. [32], the use of positively valenced smoking pictures might account for the failure to find a significant group difference between smokers and non-smokers. Indeed, in a recent study by Stippekohl and colleagues [33] no differences in the magnitude of a related cognitive bias (attention bias) between smokers and non-smokers were reported when using the same images depicting the initial phase of smoking ritual [33].

The second important finding of this study was that in contrast to non-smokers, smokers did not show an approach bias toward natural rewards. This indicates that the approach bias in smokers caused by smoking-related pictures is stronger than that caused by naturally rewarding cues, i.e. pictures of highly palatable food. This finding is in line with propositions made by the incentive sensitization theory of drug dependence [18], [31] which assumes that in drug users, drug cues acquire incentive motivational properties over time, leading to increased attention as well as approach behavior to these drug cues, and a decreased response to naturally rewarding stimuli or activities. Such cognitive biases in drug users are proposed to operate automatically and play a causal role in drug-seeking behavior and relapse, leading addicts to maintain drug-taking instead of engaging in other, less harmful and rewarding activities [16], [11], [5]. While several neuroimaging studies already showed that smokers exhibit reduced neuronal activation in response to natural rewards [19–21], to our knowledge this is the first evidence that a reduced responsiveness (i.e., a diminished approach bias in the AAT) toward natural rewards can also be measured at the behavioral level. A characteristic hallmark of addiction is a dysregulation of brain reward systems which can be expressed at the level of hedonic changes as well as an increased negative affective status [34–35]. Chronic drug use leads to anhedonia as well as an increased reward threshold for natural reinforcers during periods of acute and long-term withdrawal [36–37]. Lubman et al. [36] showed that opiate-dependent individuals show reduced reward responsiveness to natural reinforcers (across a range of measures, i.e. self-report, expressive, reflex modulation, and cortical/attentional measures) as well as an increased reward responsiveness to drug cues compared to non-drug rewards. Most importantly, subjective ratings of pleasant pictures in opiate users robustly predicted future heroin use at follow-up, even after controlling for baseline craving and heroin use. These data suggest that chronic drug users have great difficulty to stop drug use and replace this behavior by other, less harmful behaviors, e.g. switching to naturally rewarding stimuli or activities. It would be important to investigate whether smokers’ diminished approach bias for food-related stimuli is also evident for other stimuli with reinforcing properties (e.g., erotic pictures or pictures of social activities), and whether such diminished approach bias is related to an important clinical outcome, or might serve as a predictor of long-term smoking behavior.

Finally, we found a discrepancy between smokers’ and non-smokers’ explicit attitudes toward smoking and their approach biases for nicotine-related images. This is in line with dual process models of addiction [4], which assume that smoking is differentially influenced by the regulatory executive system (which is probably assessed by self-reported attitude measurements) and the appetitive/approach oriented system (which is probably reflected by approach behavior in the AAT), resulting in an imbalance between the two systems. The given discrepancy in the current study between performance in the AAT and the self-reported attitude measures may mirror this imbalance. A possible reason for this discrepancy may be that indirect tasks such as the AAT are less susceptible to individual demands or social desirability [32]. Thus, a non-smoking individual may express concerns about smoking and therefore indicate a negative subjective evaluation (i.e., attitude toward smoking), but at the same time show a positive implicit evaluation of smoking as expressed in an automatic approach bias. This shows that it is necessary to assess both the reflective system by means of direct measures, and the associative/approach-oriented system by means of more indirect tasks such as the AAT.

Recently, based on the AAT, specific training programs have been developed with the aim to change existing approach biases in addictions. For example, in a recent study by Wiers et al. [12], alcohol-dependent patients were assigned either to a training condition in which they were trained to avoid alcohol pictures and approach pictures of soft drinks, or to an AAT placebo training where no such contingency existed, or to a condition with no AAT training at all. After four sessions of approach bias retraining, patients took part in regular inpatient treatment (i.e., cognitive-behavioral therapy). The authors found that in the experimental condition, the patients’ approach bias for alcohol changed into an avoidance bias. Moreover, this change generalized to other pictures never used in the training sessions, and even to a completely other indirect task – a verbal approach-avoid IAT – which had also previously been used for the assessment of approach biases. Most importantly however, the AAT training was associated with an improved treatment outcome in patients at the one-year follow-up. In particular, patients of the experimental condition of the AAT training reported 13% less relapse relative to participants who received the control condition of the AAT training or no AAT-training at all. This effect has been replicated in a subsequent study with a larger sample of alcohol-dependent patients [16]. Contrary to the studies mentioned above, our main finding was that smokers exhibit merely a moderate approach bias for smoking-related stimuli. However, a re-training program can be aimed at not only extinguishing an existing nicotine-approach bias, but also at modifying a moderate bias as in our case into an avoidance bias for nicotine stimuli. Note that in their alcohol re-training study mentioned above, Wiers et al. [12] also failed to replicate a pre-existing alcohol approach-bias in a sample of alcohol-dependent inpatients, but were capable of changing this rather neutral approach bias for alcohol into an avoidance bias by means of a re-training AAT which affected treatment outcome at a one-year follow-up. Furthermore, it would be interesting to investigate whether such nicotine re-training would lead to a reduction in nicotine consumption and/or prevention of relapse. Such possible effects of AAT training programs on smoking behavior could be examined as a stand-alone treatment, or in combination with psychotherapeutic interventions [6].

In summary, our results have clear implications for future studies aimed to implement cognitive bias modification programs (CBM) in smokers by using a modified re-training AAT. These programs could incorporate nicotine-related cues as a category of stimuli to be avoided and stimuli corresponding to natural rewards such as palatable food, social interactions, erotic pictures or pictures of other pleasant activities, as a category of stimuli which should be approached. Such specifically designed tasks could help to reverse the “negative loop of addiction” where individuals fail to benefit from naturally rewarding properties of other stimuli and actions, but instead solely rely on stimuli associated with smoking behavior.

## Supporting Information

S1 Fig Mean AAT-Bias Scores and individual AAT-Bias Scores for nicotine-related images, images of toothbrushes, food images and neutral images, in smokers and non-smokers. Squares represent the mean scores, error bars cover +/− 1 standard error, circles represent individual bias scores of a particular person.
(TIF)

S1 Table Average scores for the eight semantic differential items concerning smoking behavior and global attitude toward smoking separated for groups. (DOCX)
