# Supplementary figures and images for: “A Cigarette a Day Keeps the Goodies Away”: Smokers Show Automatic Approach Tendencies for Smoking—But Not for Food-Related Stimuli
Source: PLoS One. 2015 Feb 18;10(2):e0116464. doi: 10.1371/journal.pone.0116464 (PMC4333198; doi:10.1371/journal.pone.0116464)

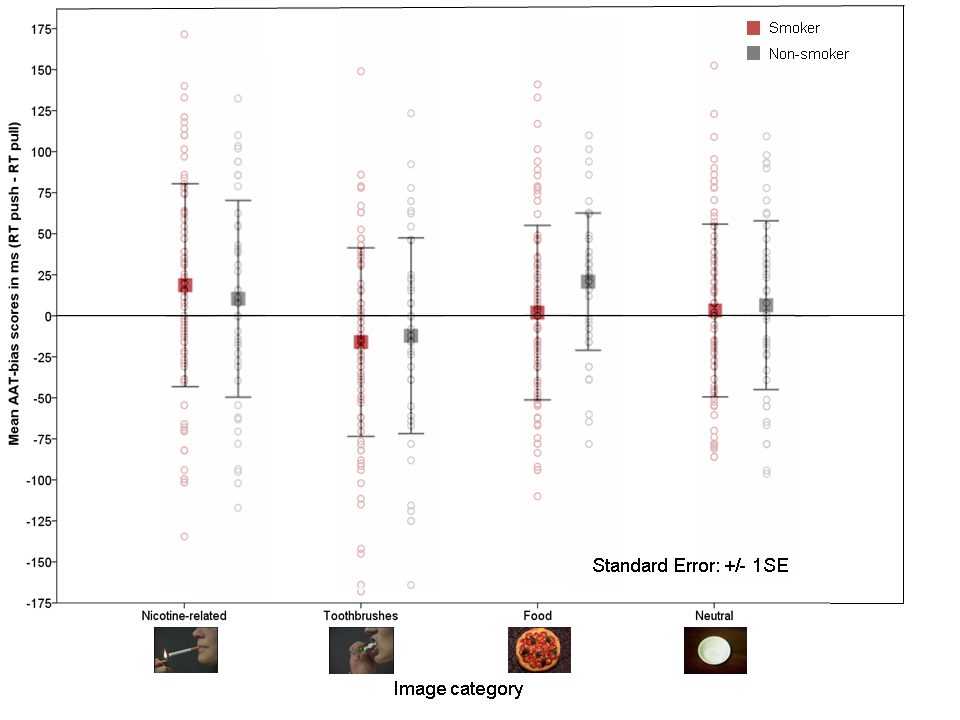

Supplement: S1 Fig — Squares represent the mean scores, error bars cover +/− 1 standard error, circles represent individual bias scores of a particular person. (TIF) [file pone.0116464.s001.tif]
