# Supplementary material for: “A Cigarette a Day Keeps the Goodies Away”: Smokers Show Automatic Approach Tendencies for Smoking—But Not for Food-Related Stimuli
Source: PLoS One. 2015 Feb 18;10(2):e0116464. doi: 10.1371/journal.pone.0116464 (PMC4333198; doi:10.1371/journal.pone.0116464)
Supplement: S1 Table — (DOCX) [file pone.0116464.s002.docx]

Table S1.

| *Group*  *Items Smokers Non-smokers Statistical Value t p-Value* | | | | |  |
| --- | --- | --- | --- | --- | --- |
| good – bad  healthy – unhealthy  sexy – unsexy  pleasant – unpleasant  harmless – harmful  sociable – unsociable  glamorous – ugly  calming – stressful | -.99(1.42)**  -2.44(.82)**  -.24(1.44)  .64(1.46)**  -2.(1.07)  1.6(1.42)**  .32(1.15)  1.67(1.14)** | -2.63(.78)**  -2.94(.24)**  -2.08(1.27)**  -2.47(.92)**  -2.69(.74)**  -.57(1.81)  -.33(2.24)  -.93(1.47)** | 56.36  16.85  55.93  184.17  16.3  60.71  5.1  133.69 | .001  .001  .001  .001  .001  .001  .2  .001 | |
| *Global attitude* | *-.16(.54)** | *-1.87(.67)*** | *271.04* | *.001* | |

*Average scores for the eight semantic differential items concerning smoking behavior and global attitude toward smoking separated for groups.*

*Note*. Standard deviations are given in parentheses; Differences between groups were analyzed with two-sample t-tests (*t*(137)); * *p* < .05 significant deviation from zero (one-sample t-test); ** *p* < .001 significant deviation from zero (one-sample t-test); all p-values are two-tailed; in case of multiple comparisons, the Bonferroni correction was used.
